# Molecular docking of alkaloid compounds with the matrix metalloproteinase 2

**DOI:** 10.6026/97320630017206

**Published:** 2021-01-31

**Authors:** JH Shazia Fathima, Jayaraman Selvaraj, Venkatacalam Sivabalan, Umapathy Vidhya Rekha, Rajagopal Ponnulakshmi, Veeraraghavan Vishnupriya, Malathi Kullappan, Radhika nalinakumari Sreekandan, Surapaneni Krishna Mohan

**Affiliations:** 1 Department of Oral and Maxillofacial Pathology, Ragas Dental College and Hospitals, Chennai, India; 2 Department of Biochemistry,Saveetha Dental College and Hospitals, Saveetha Institute of Medical and Technical Sciences, Saveetha University, Chennai - 600 077, India; 3 Department of Biochemistry, KSR Institute of Dental Sciences and Research, Thiruchengodu-637215, India; 4 Department of Public Health Dentistry, Sree Balaji Dental College and Hospital, Pallikaranai, Chennai-600 100, India; 5 Central Research Laboratory, Meenakshi Academy of Higher Education and Research (Deemed to be University), Chennai-600 078, India; 6 Department of Research, Panimalar Medical College Hospital & Research Institute, Varadharajapuram, Poonamallee, Chennai - 600 123, India; 7 Department of Clinical Skills & Simulation, Panimalar Medical College Hospital & Research Institute, Varadharajapuram, Poonamallee, Chennai - 600 123, India;; 8 Department of Biochemistry and Department of Clinical Skills & Simulation, Department of Research, Panimalar Medical College Hospital & Research Institute, Varadharajapuram, Poonamallee, Chennai - 600 123

**Keywords:** MMP-2, OSCC, alkaloids, molecular docking

## Abstract

Matrix metalloproteinase protein-2 (MMP-2) is linked to the human oral squamous cell carcinoma. Therefore, it is of interest to design new inhibitors for MMP-2 to combat the disease. Thus, we document the molecular docking features of Aristolochic acid, Cryptopleurine, Epipodophyllotoxin, and Fagaronine with MMP-2 for further consideration.

## Background

Matrix metalloproteinase protein-2 (MMP-2) is linked to the human oral squamous cell carcinoma [1-6]. Data shows the role of MMP-2 as a cancer prognostic marker [7] for prognosis [8]. Therefore, it is of interest to design new inhibitors for MMP-2 to combat the disease.

## Materials and Methods:

### Ligand Preparation:

Ligands are downloaded from the Pubchem database (Table 1 - see PDF) [9] and converted into 3D data using Pymol. The data was stored for AutoDock vina-PyRx in pdb file format. Ligand data were then saved for PyRxx in the PDBQT file format.

### Protein Preparation:

The protein PDB ID 1HOV for MMP-2 was downloaded from the RCSB protein database PDB for this study and prepared according to a standard procedure [10].

###  Molecular Docking:

The PyRx Version 0.8 [11,12] was used for molecular docking analysis. The results were visualized using Pymol.

## Results and Discussion:

**Figure 1:**
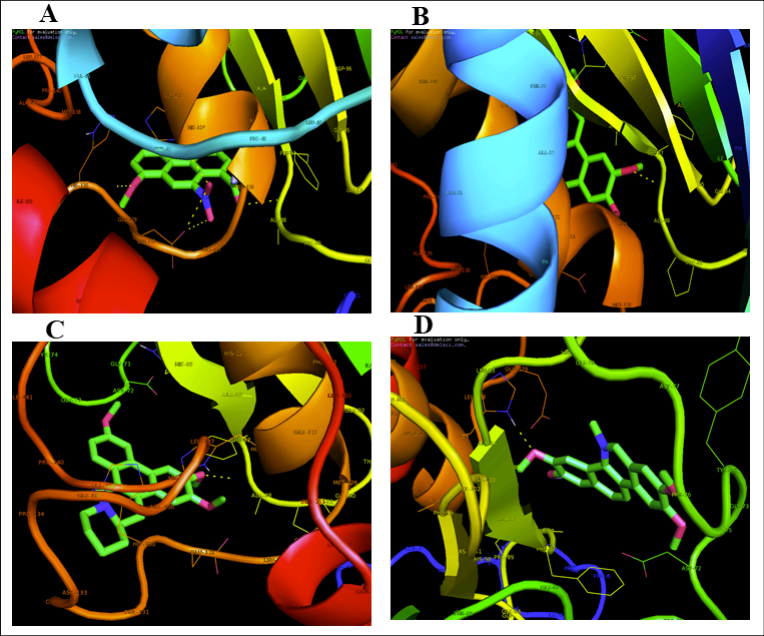
Molecular docking interaction of MMP-2 with (a) Aristolochic acid; (b) Cryptopleurine; (c) Epipodophyllotoxin; and (d) Fagaronine

Alkaloids seem to be significant chemical compounds for drug development. Alkaloids derived from natural herbs exhibit antiproliferation and antimetastasis effects on different forms of cancers. A significant component of the chemotherapy arsenal is its use for cancer care. The role of alkaloids appears to be unique in the cell cycle. Therefore, certain alkaloid compounds from various medicinal plants are used to assess their effectiveness against MMP-2 in OSCC. The top four best-docked compounds with a stronger affinity for the MMP-2 receptor were chosen, according to binding mode and molecular interaction analysis in the MMP-2 binding cavity (Table 2 - see PDF). Molecular interaction and docking measurements indicate that the lead compound Aristolochic acid offers −7.01 kcal/mol binding energy and has been shown to be more effective as an inhibitor of MMP-2 against MMP-2. Hydrogen bonding is an interaction reaction whereby the donors of hydrogen bonds and free protein and ligand acceptors sever their hydrogen bonds with water and in the protein-ligand complex form new ones. In addition to filtering unrealistic poses in docking, hydrogen-bonding formation could also be used to increase the precision of binding energy measurement. The Aristolochic acid molecular interaction analysis offers insights into their binding mode with the MMP-2 receptor and the MMP-2 amino acids that evaluate the efficacy of the docking compound. Four hydrogen bonds formed by PHE-87, HIS-124, GLU-129, and HIS-130 with bond lengths of 2.9Å, 2.4Å, 2.9Å, 2.5Å have been formed by aristolochic acid (Figure 1a). There were less than three H-bonds, suggesting the existence of favourable associations between the ligand and the receptor. The association of cryptopleurin with the protein MMP-2 was shown in Figure 1b. The amino acid residue ALA-88 formed the hydrogen bond interaction with MMP-2 at a distance of 2.5 Å. It has been calculated that cryptopleurine docking energy with MMP-2 is 6.4 Kcal / Mol. Figure 1c shows the docking conformation of Epipodophyllotoxin with MMP-2. The amino acids related to the interaction of the hydrogen bond with MMP-2 reacting with Epipodophyllotoxin were determined to be ALA-88 and the bonding wavelength of hydrogen is 2.4Å. The docking energy was computed and identified to be 6.2 Kcal/Mol for Epipodophyllotoxin with MMP-2. The docking conformation of Fagaronine with MMP-2 is shown in Figure 1d. Bonded hydrogen bond with residues HIS-124 of MMP-2 with a distance of 2.1Å is reinforced by Fagaronine with MMP-2 complex. It was observed that the binding energy for Fagaronine to MMP-2 was-6.0 Kcal / Mol.

## Conclusion

We document the molecular docking features of Aristolochic acid, Cryptopleurine, Epipodophyllotoxin, and Fagaronine with MMP-2 for consideration in the context of OSCC.
